# MiR-155 promotes proliferation of human breast cancer MCF-7 cells through targeting tumor protein 53-induced nuclear protein 1

**DOI:** 10.1186/1423-0127-20-79

**Published:** 2013-10-24

**Authors:** Chun-Mei Zhang, Jing Zhao, Hua-Yu Deng

**Affiliations:** 1 Department of Pathophysiology, School of Basic Medicine, Chongqing Medical University, 1 Medical School Road, Chongqing 400016, China

**Keywords:** MiR-155, TP53INP1 (tumor protein 53-induced nuclear protein 1), Proliferation, MCF-7 breast cancer cells

## Abstract

**Background:**

MiR-155 has emerged as an “oncomiR”, which is the most significantly up-regulated miRNA in breast cancer. However, the mechanisms of miR-155 functions as an oncomiR are mainly unknown. In this study, the aims were to investigate the effects of miR-155 on cell proliferation, cell cycle, and cell apoptosis of ERalpha (+) breast cancer cells and to verify whether TP53INP1 (tumor protein 53-induced nuclear protein 1) is a target of miR-155, and tried to explore the mechanisms of miR-155 in this process.

**Results:**

The expression of miR-155 is significantly higher in MCF-7 cells compared with MDA-MB-231 cells. Ectopic expression of TP53INP1 inhibits growth of MCF-7 cells by inducing cell apoptosis and inhibiting cell cycle progression. Overexpression of miR-155 increases cell proliferation and suppress cell apoptosis, whereas abrogating expression of miR-155 suppress cell proliferation and promotes cell apoptosis of MCF-7 cells. In addition, miR-155 negatively regulates TP53INP1 mRNA expression and the protein expression of TP53INP1, cleaved-caspase-3, -8, -9, and p21, and luciferase reporter reveals that TP53INP1 is targeted by miR-155.

**Conclusions:**

TP53INP1 is the direct target of miR-155. MiR-155, which is overexpressed in MCF-7 cells, contributes to proliferation of MCF-7 cells possibly through down-regulating target TP53INP1.

## Background

MicroRNAs (miRNAs) are a class of short (21–25 nt), on-coding RNAs, which control their target genes expression at the post-transcriptional level [1,2]. MiRNAs encoding genes are mostly transcribed by RNA polymerase II by Drosha into short hairpin RNAs, which are then exported from the nucleus, and processed by Dicer to form mature 21–25 nucleotide miRNAs, which are finally transferred to Argonaute proteins in RISC [3,4]. MiRNAs act on gene expression by binding to the 3'-untranslated region (3'UTR) of the target mRNAs, resulting in mRNA cleavage and/or translational inhibition, thereby down-regulating target proteins expression [5]. In fact, almost 30% of the protein-coding genes are under the regulation of miRNAs, and miRNAs have been implicated in the regulation of various biological functions, including cell proliferation, apoptosis, and cell differentiation [6]. Therefore, miRNA dysfunction may contribute to a variety of human diseases, including cancer [7]. MiRNAs exert their function as oncogenes or tumor suppressor genes depending on their target genes.

MiR-155, located in chromosome 21q21, is encoded with a region known as B cell integration cluster (BIC) gene, which consists of three exons within a 13 kb region [8]. The human BIC gene is activated by promoter insertion and lacks a long open reading frame [8]. MiR-155 represents a typical multifunctional miRNA, which is overexpressed in a variety of human solid tumors such as breast cancer [9-12], lung cancer [13,14], thyroid tumor [15], pancreatic cancer [16-19]. These recent clinical pathologic data indicate that miR-155 plays a crucial role in tumor development and tumor diagnosis and prognosis. The available experimental evidences indicate that miR-155 promotes some tumors growth, invasion, and metastasis through inhibits downstream targets such as SHIP1 [20], C/EBPbeta [21], and SOCS1 [11]. All these lines of evidence demonstrate that miR-155 functions as an oncomiR in human cancer.

MiR-155 has been found to be up-regulated in breast cancer and is closely related to the status of estrogen receptor (ER) and progesterone receptor (PR) [9,22], its tumorigenesis role has not yet been defined. In this study, we identified that TP53INP1 is one of the targets of miR-155 in ERalpha (+) MCF-7 cells. Furthermore, we found that overexpression of miR-155 promoted cell proliferation and inhibited apoptosis through the repression of TP53INP1 in MCF-7 cells. In addition, in this study, we indicated that miR-155 significantly repressed cell cycle and apoptosis-related gene caspase-3, -8, -9, and p21 expression. These results revealed that miR-155 is an oncomiR in breast cancer, and miR-155 may serve as a therapeutic option in breast cancer treatment.

## Methods

### Reagents

Four synthetic, chemically modified short RNA oligonucleotides: miR-155 mimics (miR-155 m, 5'-UUAAUGCUAAUCGUGAUAGGGGUCCCUAUCACGAUUAGCAUUAAUU*-*3'), miR-155 mimics negative control (miR-155 m NC, Sense: 5'-UUCUCCGAACGUGUCACGUTT-3'; Anti-sense: 5'-ACGUGACACGUUCGGAGAATT-3'), miR-155 inhibitors (miR-155i, 5'-ACCCCUAUCACGAUUAGCAUUAA-3') and miR-155 inhibitors negative control (miR-155i NC, 5'-CAGUACUUUUGUGUAGU ACAA-3') were purchased from Shanghai GenePharma Co.Ltd. The TP53INP1 overexpression plasmid and control plasmid were purchased from Wuhan Sanying Company and subcloned into pcDNA3. TP53INP1 3'UTR reporter plasmids pYr-MirTarget, pYr-MirTarget-TP53INP1-3U (pMIR-TP53INP1-3U) and pYr-MirTarget-TP53INP1-3U-M (pMIR-TP53INP1-3U-M) were purchased from Changsha Yingrun Biotechnology Co.Ltd.

### Cell culture and transfection

Human MCF-7, MDA-MB-231 cells were provided by Staff Room of Pathophysiology, Chongqing Medical University and were maintained DMEM (Gibco) or RPMI1640 (Gibco) media supplemented with 10% NBCS (Gibco, New Zealand), penicillin (100 U/ml), and streptomycin (100 μg/ml). Cells were maintained in sterile conditions at 37°C in a humidified atmosphere of 5% CO_2_. Cells were seeded into 6-well plates grown overnight and reached 30-50% confluence before transfection. Transfection of MCF-7 cells with miR-155 m (or miR-155i) or counterpart negative control using lipfectamine™ 2,000 transfection reagent (Invitrogen, USA) according to the manufacturer’s instruction. The experiments were classified into three groups, which were miR-155 m (or miR-155i) group, miR-155 m NC (or miR-155i NC) group and control (Con) group. Transfection of MCF-7 cells with TP53INP1 overexpression plasmid or control plasmid using lipfectamine™ 2,000 transfection reagent.

### RNA isolation and real-time quantitative PCR

For quantification of miR-155 and TP53INP1 mRNA by real-time PCR, total miRNA, and total RNA were isolated from cultured cells with MiRNA rapid extraction kit and RNApure rapid extraction kit (Bioteke Corporation, Beijing, China) according to the manufacturer’s instructions. Reverse transcription was performed using Moloney murine leukemia virus reverse transcriptase (Bioteke Corporation, Beijing, China). MiR-155 was reverse transcripted by looped primers with the sequence of 5'-GTCGTATCCAGTGCAGGGTCCGAGGTATTCGCACTGGATACGACACCCCT-3', while reverse transcription of TP53INP1 mRNA was performed with Oligo dT primers (Bioteke Corporation, Beijing, China). Real-time PCR was performed using the LightCycle RNA amplification kit SYBR Green (Bioteke Corporation, Beijing, China) according to the manufacturer’s protocols. U6 snRNA and *β-*actin served as an endogenous control for normalization. Real-time PCR reactions were performed on a CFX96 real-time PCR detection system from Bio-Rad Co.LTD (America), with cycle threshold values evaluated using the manufacturer’s software. The expression of miR-155, TP53INP1 mRNA, relative to U6 snRNA, *β-*actin, were calculated using the 2^-ΔΔCT^ method. The real-time PCR primers are shown in Table 1.

**Table 1:** Primers used for real-time quantitative PCR

**Gene**	**Primer sequences (5’ to 3’)**
*miR-155*	F: 5-GCGGTTAATGCTAATCGTGAT-3
	R: 5-GTGCAGGGTCCGAGGT-3
*U6*	F: 5-CTCGCTTCGGCAGCACA-3
	R: 5-AACGCTTCACGAATTTGCGT-3
*TP53INP1*	F: 5-GCACCCTTCAGTCTTTTCCTGTT-3
	R: 5-GGAGAAAGCAGGAATCACTTGTATC-3
*β-actin*	F: 5-CTGGGACGACATGGAGAAAA-3
	R: 5-AAGGAAGGCTGGAAGAGTGC-3

### Cell proliferation assay

The cell viability was evaluated by CCK8 assay according to the manufacturer’s instructions. In brief, cells were seeded into 96-well plates with 4,000 cells/well at 12 h post-transfection. Afterward 10 μl of CCK8 was added to each well, and the culture medium was incubated at 37°C for additional 3 h. Absorbance at 450 nm wavelength was detected using Eliasa. The absorbance at 450 nm shows mitochondrial activity, and it indirectly reflects living cell number, which indicates cell viability. Each experiment was performed at least in triplicate.

### Cell cycle assay

Cell cycle analysis was performed using the standard propidium iodide (PI) method. In brief, cells were trypsinized, washed with cold PBS, fixed in 70% ethanol for 24 h, and stained with PI for 30 min. Finally, the cells were analyzed by flow cytometry (FCM). Each experiment was performed at least in triplicate.

### Cell apoptosis assay

The apoptosis was analyzed by FCM. Apoptotic cells were differentiated from viable or necrotic ones by combined application of annexin V-FITC and PI. The samples were washed twice and adjusted to a concentration of 1×10^6^ cells/ml with cold PBS. 10 μl of annexin V-FITC and 10 μl PI were added into 100 μl of cell suspension, incubated for 15 min at room temperature in the dark. Finally, 400 μl of binding buffer was added to each sample without washing and analyzed using FCM. Each experiment was performed at least in triplicate.

### Western blot analysis

Protein samples were separated by 12% SDS-PAGE and transferred to PVDF membranes (Millipore). After blocked 2 h with 5% milk, the PVDF membranes were then incubated with TP53INP1 (rabbit anti-human polyclonal antibody; 1:100, Santa Cruz), pro-caspase-3 (rabbit anti-human monoclonal antibody; 1:1,000, epitomics), cleaved-caspase-3 (rabbit anti-human monoclonal antibody; 1:1,000, Beyotime), caspase-8 (rabbit anti-human monoclonal antibody; pro-caspase-8: 1:1,000, cleaved-caspase-8: 1:500, Beyotime), caspase-9 (mouse anti- human monoclonal antibody; 1:1,000, Beyotime), and p21 (mouse anti- human monoclonal antibody; 1:200, Beyotime) overnight at 4°C. Following extensive washing for a total of 45 min, membranes were incubated with secondary antibody goat anti-rabbit IgG (1:5,000, Beijing 4A Biotech Co.Ltd) and goat anti-mouse IgG (1:5,000, Beijing 4A Biotech Co.Ltd) for 2 h. After washing three times for a total of 45 min with TBST at room temperature, chemiluminescent detection was performed with the BeyoECL Plus kit (Beyotime), and membranes were exposed to Kodak XBT-1 films.

### Luciferase report assay

The pYr-MirTarget-Report plasmids for miR-155 target TP53INP1 3'UTR were constructed as pMIR-TP53INP1-3U containing the wild-type 3'UTR of TP53INP1 and as pMIR-TP53INP1-3U-M containing the mutant 3'UTR of TP53INP1. The pMIR-TP53INP1-3U vector and pMIR-TP53INP1-3U-M vector contain Firefly luciferase and Renilla luciferase. 293 cells were seeded into 96-well plates, and each was co-transfected with pMIR-TP53INP1-3U vector or pMIR-TP53INP1-3U-M vector and 50 nM miR-155 m or miR-155 m NC according to the manufacturer’s protocol. After 48 h transfection, luciferase activity was measured with the Dual Luciferase Assay system (Promega). Renilla luciferase activity was normalized to Firefly luciferase activity (Renilla LUC/ Firefly LUC). Each experiment was performed at least three times.

### Statistical analysis

Each experiment was performed in triplicate, and repeated at least three times. The data were shown as the mean ± SD, and differences were compared between 3-group using one-way ANOVA and 2-group using *t* tests. The *P* ≤ 0.05 was considered to be statistically significant.

## Results

### Differential expression of miR-155 between MCF-7 and MDA-MB-231 cells

**Figure 1:**
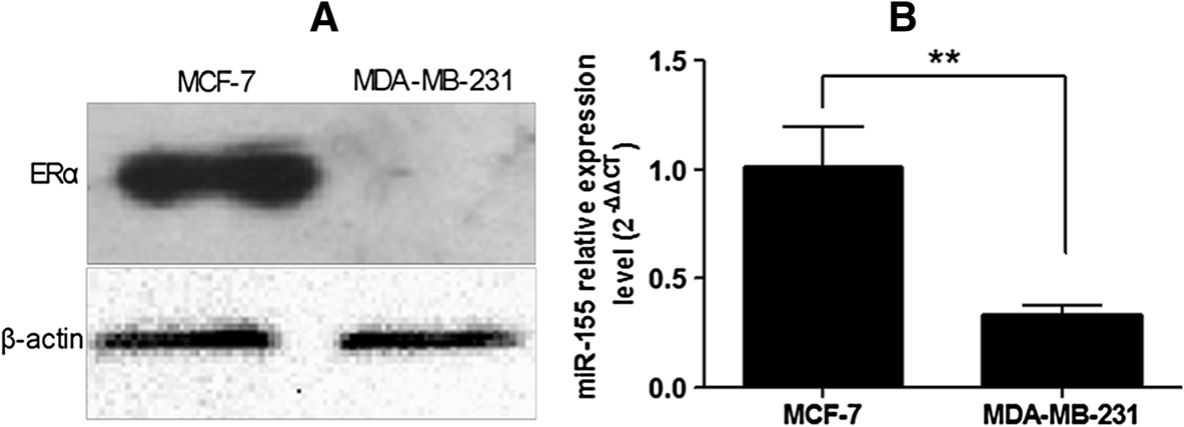
**The expression levels of ERalpha and miR-155 in MCF-7 and MDA-MB-231 cells. (A)** Expression level of ERalpha was detected by western blot of protein extracts from ERalpha (+) MCF-7 and ERalpha (−) MDA-MB-231 cells. **(B)** Differential expression of miR-155 between MCF-7 and MDA-MB-231 cells. The expression of miR-155 in MCF-7 cells was 3.05-fold that of MDA-MB-231cells. **P < 0.01. All experiments were repeated at least three times.

ERalpha expression was monitored by western blot analysis, using as control extracts from ERalpha (−) MDA-MB-231 cells (Figure 1A). In our study, we found that miR-155 expression was significantly higher in MCF-7 cells with ERalpha (+) compared with MDA-MB-231 cells with ERalpha (−) using real-time PCR analysis. As shown in Figure 1B, the expression of miR-155 in MCF-7 cells was 3.05-fold that of MDA-MB-231 cells and the difference had statistical significance.

### Ectopic expression of TP53INP1 inhibits growth of MCF-7 cells by inducing cell apoptosis and inhibiting cell cycle progression

**Figure 2:**
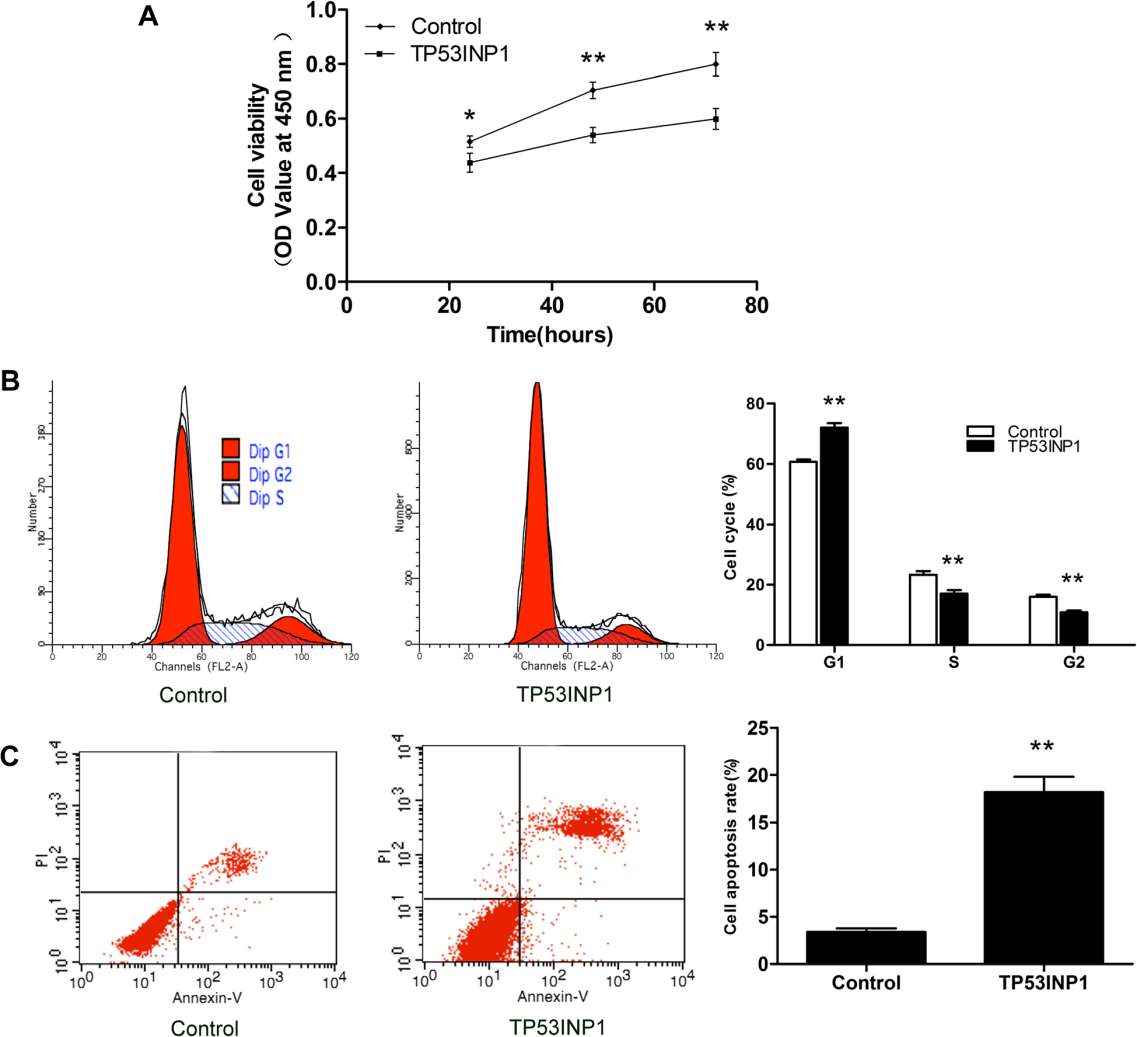
**TP53INP1 inhibits MCF-7 cell proliferation and promotes cell apoptosis. (A)** CCK8 analysis of cell viability after transfection with TP53INP1 overexpression plasmid or control plasmid. *P < 0.05 and **P < 0.01 versus control. **(B)** FCM analysis of cell cycle after transfection with TP53INP1 overexpression plasmid or control plasmid. **P < 0.01 versus control. **(C)** FCM analysis of cell apoptosis rate after transfection with TP53INP1 overexpression plasmid or control plasmid. **P < 0.01 versus control. Data are presented as mean ± SD. All experiments were repeated at least three times.

MCF-7 cells were transfected with TP53INP1 overexpression plasmid or control plasmid, and CCK8 assay showed there was a significant reduction in the growth of TP53INP1-expressing cells compared to those transfected with the control plasmid (Figure 2A).

To elucidate the mechanism of growth inhibition of TP53INP1-expressing MCF-7 cells, we analyzed their cell cycle and cell apoptosis rate. Overexpression of TP53INP1 resulted in a significant increase in the percentage of cells in the G1 phase indicating a G1 cell cycle arrest (Figure 2B). FCM assay showed that overexpression of TP53INP1 increased cell apoptosis rate compared with the control plasmid (Figure 2C). These data revealed that TP53INP1 inhibited the growth of MCF-7 cells by inducing cell apoptosis and inhibiting cell cycle progression.

### TP53INP1 is a target of miR-155 in breast cancer cells

**Figure 3:**
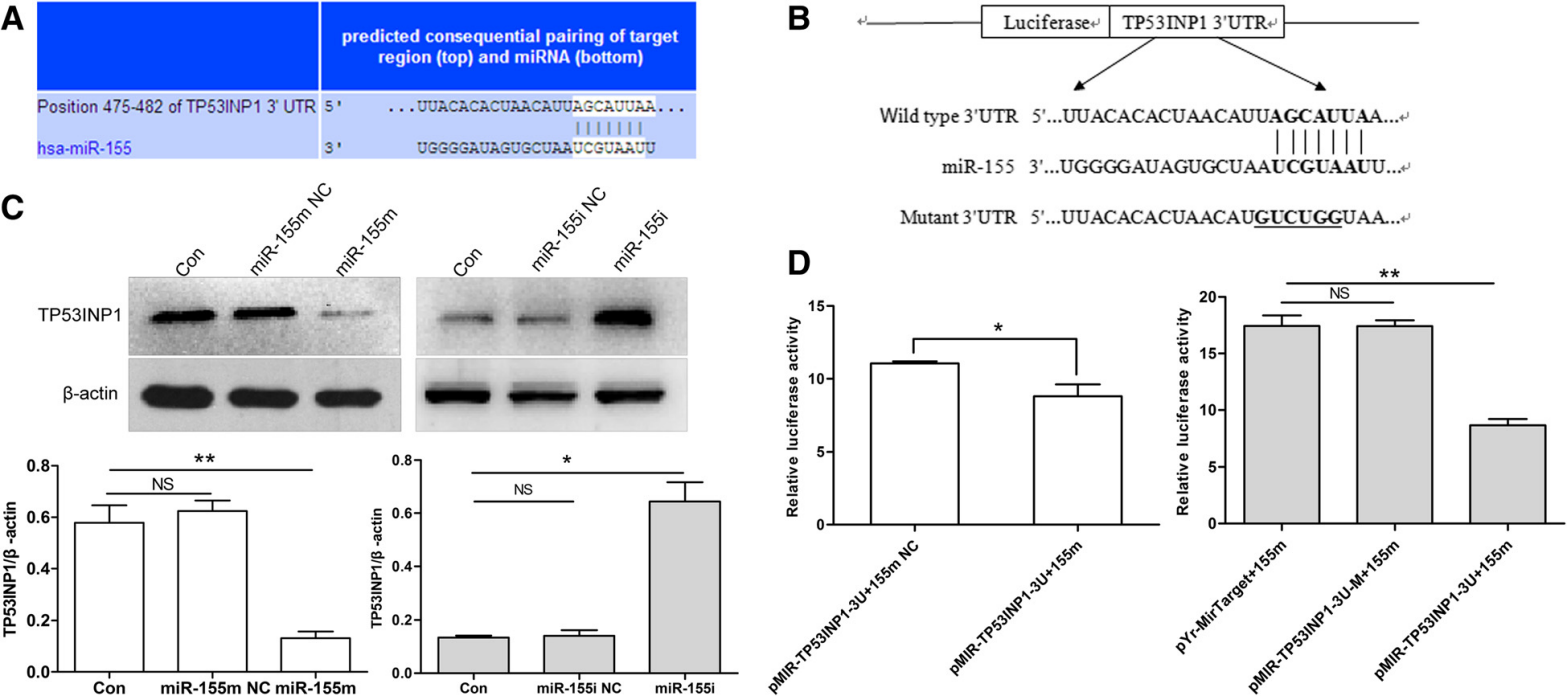
**TP53INP1 is a target of miR-155 in MCF-7 cells. (A)** Putative binding sites of miR-155 in the TP53INP1 3'UTR (white sequences) predicted by TargetScan. **(B)** Sketch of the construction of pMIR-TP53INP1-3U or pMIR-TP53INP1-3U-M vectors. The mutant binding site is underlined. **(C)** TP53INP1 protein level was measured by western blot at 72 h post-transfection. Data are presented as mean ± SD. **P < 0.01 and *P < 0.05 versus Con, miR-155 m NC or miR-155i NC. **(D)** miR-155 m down-regulated luciferase activity controlled by wild-type TP53INP1 3'UTR, but did not affect luciferase activity controlled by mutant TP53INP1 3'UTR. *P < 0.05 versus pMIR-TP53INP1-3U+155 m NC and **P < 0.01 versus pYr-MirTarget+155 m, pMIR-TP53INP1-3U-M+155 m. All experiments were repeated at least three times.

To study the mechanisms and carcinogenic function of miR-155 on the development of breast cancer, TargetScan, Pictar-Vert, and microRNA.Org were using for this purpose. In combinational predicted with the three softwares, we found that TP53INP1 was a potential target of miR-155. The TP53INP1 3'UTR carries a binding site for miR-155 (Figure 3A), suggesting that TP53INP1 might be a direct target of miR-155. Therefore, we determined that whether overexpression of miR-155 led to down-regulation of TP53INP1 expression in human breast cancer cells.

**Figure 4:**
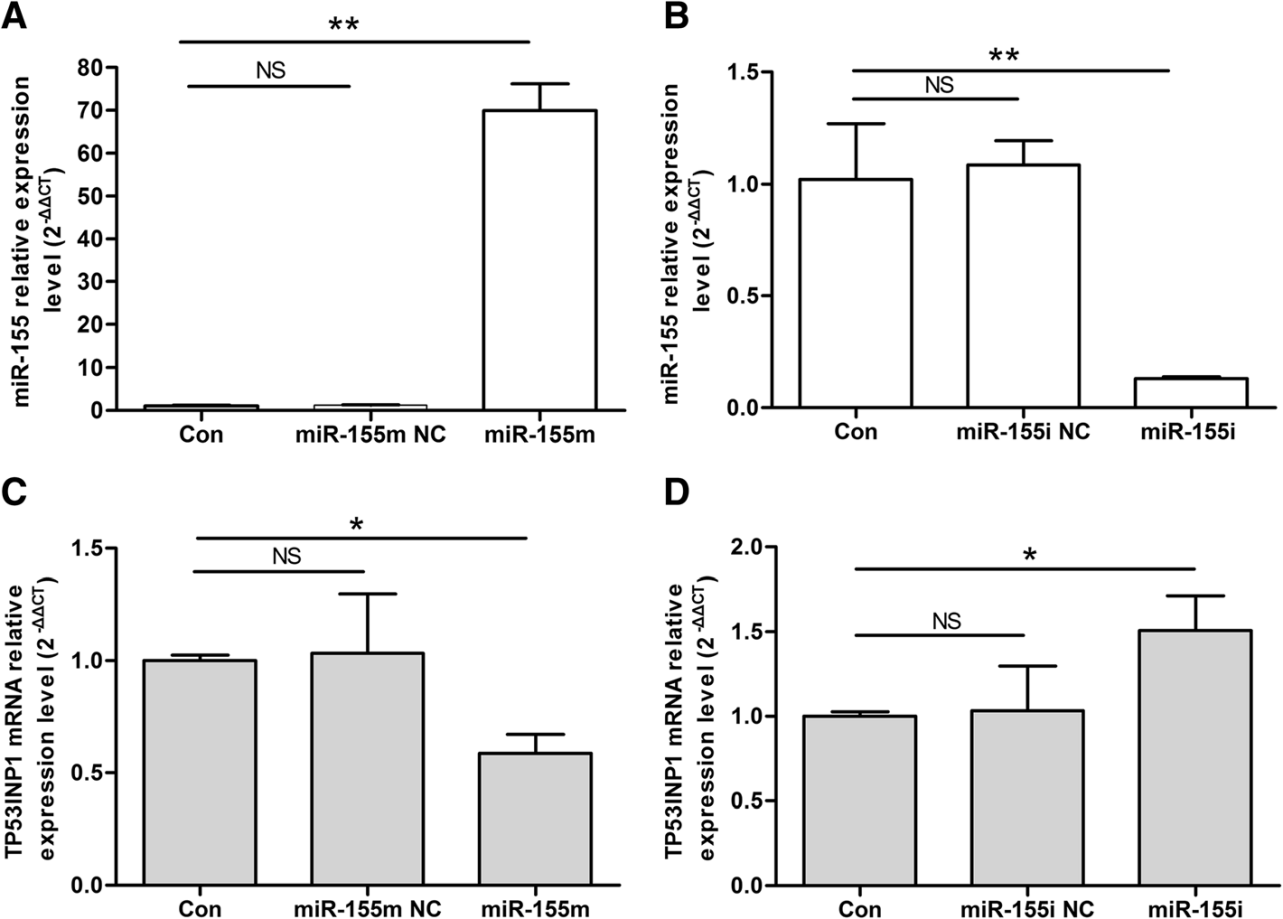
**Analysis of the expression levels of miR-155 and TP53INP1 mRNA after differential treatment. (A)** Expression level of miR-155 after transfection with miR-155 m. Data are presented as mean ± SD. **P < 0.01 versus Con, miR-155 m NC. **(B)** Expression level of miR-155 after transfection with miR-155i. **P < 0.01 versus Con, miR-155i NC. **(C)** Expression level of TP53INP1 mRNA after transfection with miR-155 m. *P < 0.05 versus Con, miR-155 m NC. **(D)** Expression level of TP53INP1 mRNA after transfection with miR-155i. *P < 0.05 versus Con, miR-155i NC. All experiments were repeated at least three times.

Real-time quantitative PCR analyses showed that the TP53INP1 mRNA level was down-regulated by ~45% when miR-155 was overexpression ~69-fold (Figure 4A, 4C). In contrast, the TP53INP1 mRNA level was up-regulated by ~1.5-fold when miR-155 was down-regulated by ~87% (Figures 4B, 4D). Moreover, western blot assay indicated that transfection with miR-155 m led to nearly 78% down-regulation of TP53INP1 in MCF-7 cells compared with Con or miR-155 m NC group, whereas treatment with miR-155i induced 359% increase of TP53INP1 compared with Con or miR-155i NC group (Figure 3C). To understand these negative regulate relationship between miR-155 and TP53INP1, we constructed plasmids containing wild-type or mutant 3'UTR of TP53INP1 fused to the luciferase gene (Figure 3B). The wild-type or mutant plasmid was co-transfected into 293 cells with miR-155 m or miR-155m NC. As shown in Figure 3D, miR-155 significantly decreased the luciferase activity of wild-type 3'UTR of TP53INP1, whereas the luciferase activity with mutant 3'UTR of TP53INP1 was not repressed by miR-155, suggesting that miR-155 could directly bind to the 3'UTR of TP53INP1. Taken together, these findings showed that TP53INP1 is a direct target of miR-155 in breast cancer cells and mRNA degradation is involved in miR-155-suppressing TP53INP1.

### MiR-155 promoted cell proliferation and inhibited cell apoptosis of MCF-7 cells

**Figure 5:**
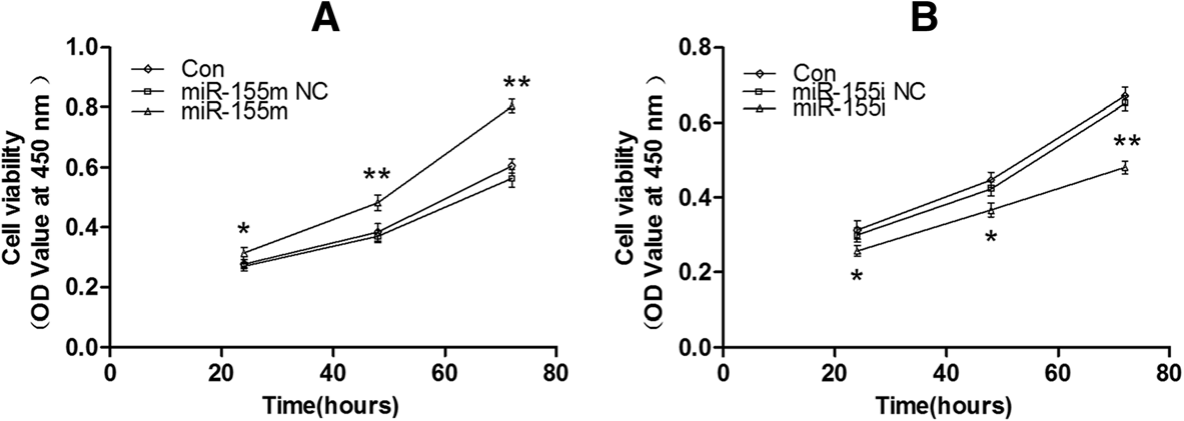
**Cell proliferation of MCF-7 cells after differential treatment. (A)** CCK8 analysis of cell viability after transfection with miR-155 m at differential times. *P < 0.05 and **P < 0.01 versus Con, miR-155 m NC. **(B)** CCK8 analysis of cell viability after transfection with miR-155i at differential times. *P < 0.05 and **P < 0.01 versus Con, miR-155i NC. The OD value reflects cell viability indirectly. All experiments were repeated at least three times.

**Figure 6:**
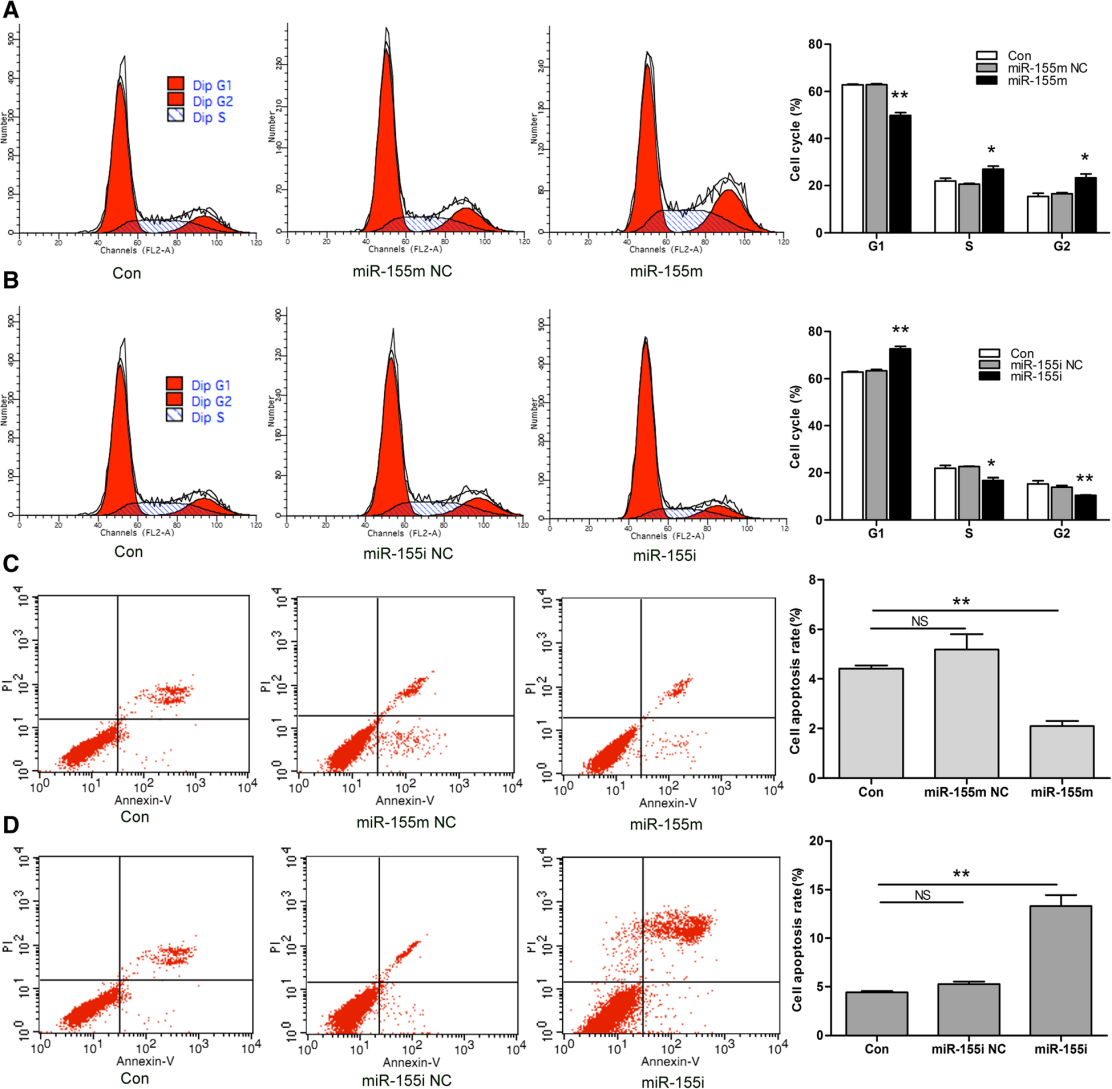
**Cell cycle and cell apoptosis rate of MCF-7 cells after differential treatment. (A)** FCM analysis of cell cycle after transfection with miR-155 m. **(B)** FCM analysis of cell cycle after transfection with miR-155i. **(C)** FCM analysis of cell apoptosis rate after transfection with miR-155 m. **(D)** FCM analysis of cell apoptosis rate after transfection with miR-155i. Data are presented as mean ± SD. **P<0.01 and *P < 0.05 versus Con, miR-155 m NC or miR-155i NC. All experiments were repeated at least three times.

To investigate the biologic effects of miR-155, cell proliferation, cell cycle, and cell apoptosis were measured after differential transfection. As shown in Figure 5A, treatment with miR-155 m significantly promoted the growth of MCF-7 cells compared with Con or miR-155 m NC. MiR-155i, but not the miR-155i NC significantly inhibited the growth of MCF-7 cells compared with Con (Figure 5B). Next, we detected the effect of miR-155 on cell cycle, found that miR-155 significantly increased S and G2 phase population, but had more profound effect on G1 phase population compared with Con or miR-155 m NC (Figure 6A). G1 phase population of MCF-7 cells transfected with miR-155i was increased, and S and G2 phase population was reduced compared with Con or miR-155i NC (Figure 6B). Moreover, FCM assay was performed to detect the effect of miR-155 on cell apoptosis of MCF-7 cells. As shown in Figures 6C, 6D, transfected with miR-155 m could significantly reduce cell apoptosis rate of MCF-7 cells compared with Con or miR-155 m NC, and transfected with miR-155i increased cell apoptosis rate compared with Con or miR-155i NC. These data suggested that miR-155 might function as an oncogene in MCF-7 cells.

### MiR-155 negatively regulated the protein expression of cleaved-caspase-3, -8, -9, and p21 in MCF-7 cells

**Figure 7:**
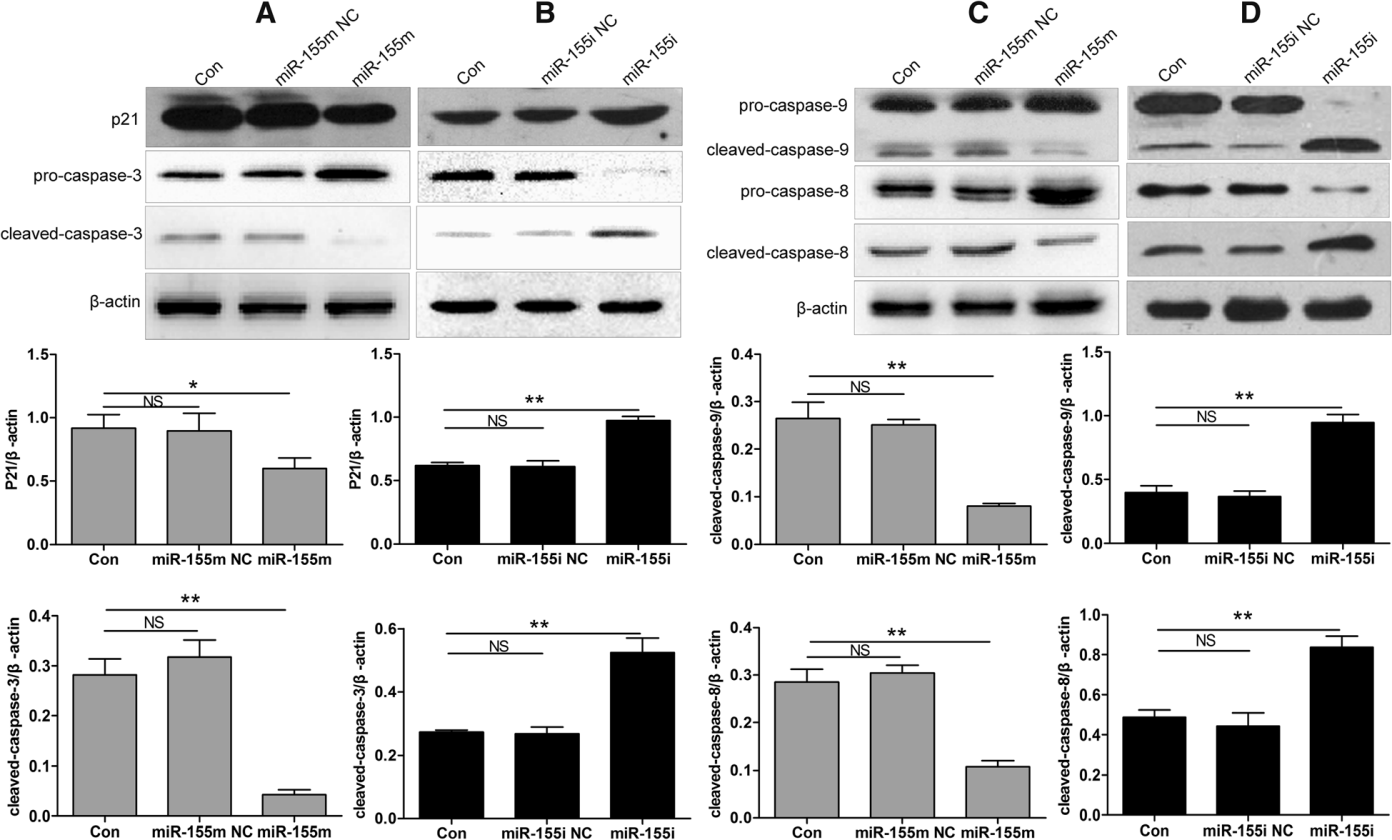
**MiR-155 negatively regulated protein expression of cleaved-caspase-3, -8, -9, and p21 in MCF-7 cells. (A)** Protein expression levels of p21 and cleaved-caspase-3 after transfection with miR-155 m. **(B)** Protein expression levels of p21 and cleaved-caspase-3 after transfection with miR-155i. **(C)** Protein expression levels of cleaved-caspase-8, -9 after transfection with miR-155 m. **(D)** Protein expression levels of cleaved-caspase-8, -9 after transfection with miR-155i. The protein expression levels of cleaved-caspase-3, -8, -9, and p21 were detected by western blot and normalized to that of *β-*actin. Data are presented as mean ± SD. **P < 0.01 and *P < 0.05 versus Con, miR-155 m NC or miR-155i NC. All experiments were repeated at least three times.

To verify the role of miR-155 in protein expression of caspase-3, -8, -9, and p21, MCF-7 cells were transfected with miR-155 m or miR-155i. Treatment with miR-155 m induced 87%, 65%, 68%, and 33% reduced in cleaved-caspase-3, -8, -9, and p21 compared with Con or miR-155 m NC, whereas transfection with miR-155i led to nearly 97%, 90%, 159%, and 60% up-regulation of cleaved-caspase-3, -8, -9, and p21 compared with Con or miR-155i NC (Figure 7). These data suggested that cell cycle and apoptosis-related protein caspase-3, -8, -9, and p21 were possible the potential downstream genes of TP53INP1.

## Discussion

The mechanisms of miR-155 functions as an oncomiR are largely unknown. Our study here was to investigate the effects of miR-155 on cell proliferation, cell cycle and cell apoptosis of ERalpha (+) breast cancer cells and to verify whether TP53INP1 is a target of miR-155, and tried to explore the mechanisms of miR-155 in this process.

In our study, we found that miR-155 expression was significantly higher in ERalpha (+) MCF-7 cells than ERalpha (−) MDA-MB-231 cells. Moreover, we observed that up-regulation of miR-155 significantly stimulated cell growth and inhibited cell apoptosis in MCF-7 cells, which was consistent with results that blocking miR-155 using miR-155 inhibitors inhibited cell growth and promoted cell apoptosis. But, how miR-155 might functions inside cells accounting for the effects of miR-155 on biologic behavior of MCF-7 cells. To study the mechanisms and carcinogenic function of miR-155 on the development of breast cancer, TargetScan, Pictar-Vert, and microRNA.Org were using for this purpose. In combinational predicted with the three softwares, we found that TP53INP1 was a potential target of miR-155. Therefore, we focused our attention on TP53INP1 signal pathways.

The main pathway in human breast cancer involve p53, which acts as a multifunctional transcription factor, exerts its tumor suppressor function mainly through transcriptional induction of target genes involved in several processes, including cell cycle checkpoints and apoptosis [23]. TP53INP1, one of the p53 target genes, is a p53-inducible cell stress response gene [24,25] located on the chromosome 8q22 [26]. Its expression is dependent on the activation of wide-type p53 [27]. Originally, TP53INP1 expression is strongly induced in mice with acute pancreatitis in vivo [24], and also in several cell lines under exposure to various stress agents in vitro [25,27,28]. Overexpression of TP53INP1, which is a tumor suppressor gene, induces cell cycle arrest as well as enhances the p53-mediated apoptosis [27]. In our study, we have also showed that ectopic expression of TP53INP1 inhibited growth of MCF-7 cells by inducing cell apoptosis and inhibiting cell cycle progression. Moreover, recent findings have shown a significant reduction of the expression of TP53INP1 during the development of breast cancer [29], stomach cancer [30], and pancreas cancer [18]. TP53INP1 expression was investigated immunohistochemically in 81 cases of breast carcinoma, compared with normal breast tissue, decreased TP53INP1 expression was found in 45 cases (55.6%) [29]. The expression level of TP53INP1 was inversely linked to high histological grade, tumor size, positive lymph node metastasis, and aberrant p53 expression [29]. Besides, it had been reported that TP53INP1-positive rate decreased with the progression of gastric cancer; and TP53INP1 protein negativity was significantly associated with aggressive pathological phenotypes of gastric cancer [30,31]. These observations suggest that TP53INP1 plays a crucial role in suppression of tumor progression including breast cancer through its anti-proliferative and pro-apoptotic activities. Our data showed that overexpression of miR-155 not only led to the down-regulation of TP53INP1 but also inversed the effect of TP53INP1 by promoting cell proliferation and suppressing cell apoptosis. Restoration of TP53INP1 by knockdown of miR-155 using miR-155 inhibitors was accompanied by decreased cell proliferation and enhanced cell apoptosis. The conclusion that miR-155 negatively regulated the protein expression of TP53INP1 was further supported by data showing that miR-155 significantly decreased the relative luciferase activity of TP53INP1 3'UTR. MiR-155 can directly repress TP53INP1 expression through binding to the binding sites in the 3'UTR of TP53INP1, thereby negatively regulating TP53INP1 function. Thus, we have reasons to believe that TP53INP1 is possible the target gene of miR-155 in MCF-7 cells.

Apoptosis is a process of programmed cell death that plays a crucial role in both normal growth and maintaining cellular homeostasis, and regulated by numerous signaling pathways [32]. Dysregulated apoptosis is caused by the changes in the expression and activation of key apoptotic regulators [33]. The caspase family of cysteine proteases plays a key role in coordinating the stereotypical events that occur during apoptosis. Caspases are classified into two groups according to their structure and function: the upstream initiator, such as caspase-8, -9, and −10, and the downstream executioner, such as caspase-3, -6, and-7 [34]. Cell cycle arrest is closely linked to apoptosis. P21 is cyclin-dependent kinase inhibitor located in p53 gene downstream, and plays a key role in cell cycle G0/G1 arrest. Our data showed that down-regulation of TP53INP1 by miR-155 correlated with down-regulation of cleaved-caspase-3, -8, -9, and p21. It indicated that TP53INP1 regulates cell cycle and induces cell apoptosis may be by down-regulating p21 and activating caspase way.

## Conclusions

In summary, our researches suggest that miR-155 functions as an oncomiR by targeting TP53INP1 and contributes to the control of cell survival and growth in breast cancer cells. Tumor suppressor gene TP53INP1 is negatively regulated by miR-155 and mediates miR-155 functions in inducing cell proliferation and inhibiting cell apoptosis. Therefore, TP53INP1 is identified to be the direct target of miR-155. The findings may be providing a new therapeutic strategy for breast cancer.

## Abbreviations

miRNA: MicroRNA; RISC: RNA-induced silencing complex; 3'UTR: 3'-untranslated region; BIC: B cell integration cluster; SHIP1: Src homology 2 domain-containing inositol-5-phosphatase 1; C/EBPbeta: CCAAT enhancer-binding protein beta; SOCS1: Suppressor of cytokine signaling 1; ER: Estrogen receptor; PR: Progesterone receptor; TP53INP1: Tumor protein 53-induced nuclear protein 1.

## Competing interests

The authors declare that they have no competing interests.

## Authors’ contributions

CMZ and HYD designed experiments. CMZ performed major experiments and data analysis. CMZ and HYD drafted manuscript. JZ provided financial support. All authors read and approved the final manuscript.
